# Methionine-Restricted C57BL/6J Mice Are Resistant to Diet-Induced Obesity and Insulin Resistance but Have Low Bone Density

**DOI:** 10.1371/journal.pone.0051357

**Published:** 2012-12-07

**Authors:** Gene P. Ables, Carmen E. Perrone, David Orentreich, Norman Orentreich

**Affiliations:** Orentreich Foundation for the Advancement of Science, Cold Spring-on-Hudson, New York, United States of America; University of Valencia, Spain

## Abstract

Dietary methionine restriction (MR) extends lifespan, an effect associated with reduction of body weight gain, and improvement of insulin sensitivity in mice and rats as a result of metabolic adaptations in liver, adipose tissue and skeletal muscle. To test whether MR confers resistance to adiposity and insulin resistance, C57BL/6J mice were fed a high fat diet (HFD) containing either 0.86% methionine (control fed; CF) or 0.12% methionine (methionine-restricted; MR). MR mice on HFD had lower body weight gain despite increased food intake and absorption efficiency compared to their CF counterparts. MR mice on HFD were more glucose tolerant and insulin sensitive with reduced accumulation of hepatic triglycerides. In plasma, MR mice on HFD had higher levels of adiponectin and FGF21 while leptin and IGF-1 levels were reduced. Hepatic gene expression showed the downregulation of *Scd1* while *Pparg*, *Atgl*, *Cd36*, *Jak2* and *Fgf21* were upregulated in MR mice on HFD. Restriction of growth rate in MR mice on HFD was also associated with lower bone mass and increased plasma levels of the collagen degradation marker C-terminal telopeptide of type 1 collagen (CTX-1). It is concluded that MR mice on HFD are metabolically healthy compared to CF mice on HFD but have decreased bone mass. These effects could be associated with the observed increase in FGF21 levels.

## Introduction

Rats and humans become obese with advancing age and obesity increases their susceptibility to insulin resistance which could lead to type 2 diabetes [1]. Although the onset of obesity and insulin resistance is multifactorial, animal models have been useful to identify mechanisms involved in the onset of these two disorders [2], [3]. One such model uses C57BL/6J mice, which have increased weight gain, fasting glucose and insulin, and reduced glucose tolerance and insulin sensitivity when fed a high fat diet (HFD) [3]. This HFD mouse model is also used to investigate events involved in the development of hepatic steatosis [4].

Dietary methionine restriction (MR) in rodents extends lifespan and protects from visceral fat mass accretion while maintaining normal insulin levels [5], [6]. Characteristic of MR rodents are higher levels of plasma adiponectin and lower levels of leptin, corresponding with the effects of MR on adiposity [5], [7]. Rodents fed MR diet are also insulin sensitive as demonstrated by insulin tolerance tests, effects associated with the downregulation of hepatic stearoyl-CoA desaturase (*Scd1*) gene expression and the upregulation of peroxisome proliferator-activated receptor gamma (*Pparγ*) suggesting decreased fatty acid synthesis and increased β-oxidation of fatty acids [7]. The MR effects in liver together with metabolic adaptations in white adipose tissue resulting in the futile cycling of fatty acids [8] led us to hypothesize that MR mice fed a HFD are protected from developing obesity and insulin resistance. Therefore, the focus of this study was to characterize the effects of MR on HFD-fed mice.

These studies showed that MR mice on HFD were protected from diet-induced obesity, type 2 diabetes and hepatic steatosis with a concomitant decrease in bone mass. One possibility is that bone mass loss in MR mice on HFD is associated with increased levels of FGF21, a hepatokine shown to reverse obesity and type 2 diabetes [9] via *Pparγ* signaling mechanisms [10]. Because the effect of FGF21 on *Pparγ* promotes bone loss [11], it is suggested that FGF21 may be involved in bone mass loss in MR mice on HFD.

## Research Design and Methods

### Animal Care

All studies were approved by the Institutional Animal Care and Use Committee of the Orentreich Foundation for the Advancement of Science, Inc (Permit Number: 0511MB). Seven week-old male C57BL/6J mice (Stock number 000664) purchased from the Jackson Laboratories (Bar Harbor, ME) were housed in a conventional animal facility maintained at 20±2°C, 50±10% relative humidity and a 12 h light: 12 h dark photoperiod. Food and water (pH 2.8) were provided *ad libitum*. Upon arrival, the mice were acclimatized for one week and fed Purina Lab Chow # 5001. After 1 week, the mice were fed either an isocaloric HFD (5.3 kcal/gm) consisting of 14% kcal protein, 26% kcal carbohydrate and 60% kcal fat (Research Diets, New Brunswick, NJ) or low fat diet (LFD, 3.9 kcal/gm) consisting of 14% kcal protein, 76% kcal carbohydrate and 10% kcal fat and then randomly separated into 0.86% methionine (control-fed; CF) or 0.12% methionine (methionine-restricted; MR) diets (Table 1 and Table S1). The 0.12% methionine-containing diet differs from the methionine-choline deficient diet used to induce steatohepatitis, which is completely depleted with methionine [12], [13]. Body weights and food consumption were monitored twice a week for the duration of the study. Cumulative food intake was measured by combining the average daily food intake of each mouse. At the end of the study, the animals were fasted for 4 hours (h) at the beginning of the light cycle to establish physiological baseline and then sacrificed. Blood was collected from the retro-orbital plexus and plasma was collected, flash frozen and stored at −80°C until analyzed. Perigonadal fat pads, liver, heart, spleen, kidneys and femur bones were harvested, flash frozen and stored at −80°C until processed.

**Table 1:** Diet composition of HFD-fed CF and MR mice.

Ingredients (gm)	60% Fat
L-Arginine	11.2
L-Histidine-HCl-H2O	3.3
L-Isoleucine	8.2
L-Leucine	11.1
L-Lysine	14.4
DL-Methionine	1.2 (8.6)
L-Phenylalanine	11.6
L-Threonine	8.2
L-Tryptophan	1.8
L-Valine	8.2
L-Glutamic Acid	34.4 (27)
Glycine	23.3
Corn Starch	0.0
Maltodextrin	56.8
Dextrose	50.0
Sucrose	150.0
Lard	219.0
Corn Oil	46.0
Minerals	35.0
Vitamins	10.0
Choline Bitartrate	2.0

High fat diets were purchased from Research Diets, Inc., New Brunswick, NJ. Control-fed (CF) on HFD catalog number: A11051306 and methionine-restricted (MR) on HFD catalog number: A11051305. Numbers in parenthesis are levels of DL-methionine and L-glutamic acid in the CF diet.

### Blood Biochemical Tests

Enzyme-linked immunosorbent assay (ELISA) kits were used to measure insulin (ALPCO Diagnostics, Salem, NH), apo B (Kamiya Biomedical Co., Seattle, WA), homocysteine (Cosmo Bio USA Inc., Carlsbad, CA), leptin, IGF-1, adiponectin (R&D Systems, Minneapolis, MN), FGF21 (Millipore Corp., Billerica, MA), N-terminal propeptide of type 1 procollagen (P1NP) and C-terminal telopeptide of type 1 collagen (CTX-1) (Immunodiagnostic Systems, Fountain Hills, AZ). Colorimetric assays were used to determine plasma triglycerides (TG) and total cholesterol (TC) (Thermo Electron Corp.), LDL and HDL (EnzyChrom™ BioAssay Systems, Hayward, CA), and free-fatty acids (FFA) (Wako Chemicals USA, Inc., Richmond, VA). Blood glucose was measured using an Abbott® Freestyle glucometer and glucose strips. Plasma alanine aminotransferase (ALT) and aspartate aminotransferase (AST) were measured using a Beckman Synchron CX5 system.

### Absorption Efficiency (AE)

Absorption efficiency of the diet by the mice was measured over a 24 h period as described previously [14] with slight modifications. At the beginning of the light cycle, mice were transferred to cages with a minimum amount of bedding; they were returned to their original cages on the following day. The amount of feces excreted by each mouse over 24 h was measured. Food intake for the 24 h study was also measured. Percent AE (AE %) was determined by the measurements from the 24 h food intake (g) minus the fecal mass (g) divided by the 24 h food intake (g).

### Glucose Metabolism Experiments

For each experiment, the mice were fasted for 6 h with free access to water. For intraperitoneal (IP) glucose tolerance tests (GTT), the mice were injected with 10% glucose (D-glucose, Sigma, St. Louis, MO) in 0.9% saline (Teknova, Hollister, CA) at a 1 mg/kg dose. For IP insulin tolerance tests (ITT), the mice were injected with 100 mU of insulin (Humulin-R, Lilly, Indianapolis, IN) in 0.9% saline solution at a 0.5 U/kg dose. For IP pyruvate tolerance tests (PTT), the mice were injected with 20% sodium pyruvate (Sigma) dissolved in PBS at a 1 g/kg dose. The mice were bled from a tail clip. Blood glucose was measured before injection (time 0) and 15, 30, 60, 90 and 120 min after injection using a handheld glucometer. The homeostasis model for insulin resistance (HOMA-IR) was calculated from the fasting blood glucose (mmol/L) × fasting plasma insulin (µU/ml) divided by 22.5.

### Histological Analysis

For hematoxylin and eosin (H & E) staining, liver tissue samples were fixed in 10% formalin solution (Thermo Scientific), paraffin embedded and subsequently sectioned at 5 µm. For neutral lipid staining using Oil red O, liver tissue was embedded in Tissue-tek optimal cutting temperature compound (Sakura Finetek), sectioned at 5 µm and counterstained for H & E. Sections for both stains were photographed at ×100 magnification.

### Hepatic Lipid Measurements

Hepatic lipids were extracted using a modification of the chloroform/methanol method described previously [15], [16]. Briefly, 100 mg of liver tissue was homogenized in 5 ml of 1 M NaCl and extracted twice in a 2∶1 (v/v) chloroform:methanol solution. The extract was dried under nitrogen gas and resuspended in 1 ml of 2% Triton X-100 solution. Hepatic TG, TC and FFA were measured colorimetrically as described above.

### Gene Expression Analysis

Total hepatic RNA was isolated using Qiagen’s RNeasy kits. RNA concentration and quality were determined using a Nanodrop (Thermo Scientific, Wilmington, DE). cDNA was prepared with the High-Capacity cDNA Reverse Transcription Kit (Life Technologies, Carlsbad, CA) in a Perkin-Elmer GeneAmp PCR System 9600 as described previously [7]. Quantitative real-time PCR (qPCR) was conducted in a StepOnePlus Real-Time PCR System using commercially available TaqMan primer-probe sets (Life Technologies, Carlsbad, CA) (Table 2). Gene expression was assessed by the comparative CT (ΔΔCT) method with β-actin as the reference gene.

**Table 2:** Taqman primer-probe sets (Life Technologies, Carlsbad, CA) used for hepatic gene expression from HFD-fed CF and MR mice.

Gene Symbol	Gene Name					ABI ID Number
*Atgl*	Patatin-like phospholipase domain containing 2					Mm00503040_m1
*Cd36*	CD36 antigen					Mm01135198_m1
*Dgat1*	Diacylglycerol O-acyltransferase 1					Mm00515643_m1
*Dgat2*	Diacylglycerol O-acyltransferase 2					Mm01273905_m1
*Fas*	Fatty acid synthase					Mm00662319_m1
*Fgf21*	Fibroblast growth factor 21					Mm00840165_g1
*G6Pase*	Glucose 6-phophatase					Mm00839363_m1
*Hsl*	Hormone sensitive lipase					Mm00495359_m1
*Jak2*	Janus kinase 2					Mm01208489_m1
*Pepck*	Phosphoenolpyruvate carboxykinase					Mm 01247058_m1
*Ppara*	Peroxisome proliferator activated receptor alpha					Mm00440939_m1
*Pparg*	Peroxisome proliferator activated receptor gamma					Mm01184322_m1
*Scd1*	Stearoyl-Coenzyme A desaturase 1					Mm01197142_m1
*Srebf1*	Sterol regulatory element-binding protein 1					Mm00550338_m1
*Stat5a*	Signal transducer and activator of transcription 5A					Mm00839861_m1

### Animal Measurements

Under light isoflurane anesthesia, length measurements were made from the tip of the nose and the base of the tail of each mouse. Body mass index (BMI) was calculated as the body weight (g) divided by the square of the anal-nasal length (cm) [17]. For femur morphometry, the bone was dissected from the soft tissue and the length and the diameters of the midshaft, anteroposterior, mediolateral, and third trochanter were measured using a caliper ruler. Bone mineral density (BMD) and bone mineral content (BMC) were measured using a PIXImus dual-energy X-ray absorptiometry (DEXA) instrument and software version 1.46 (GE Lunar, Madison, WI) from the Diabetes and Endocrinology Research Center (DERC) at Columbia University Medical Center, New York, NY.

### Rotarod Experiments

Rotarod experiments were conducted as reported previously [18], but with some modifications. Briefly, for accelerated rotarod experiments, the mice were allowed to familiarize with the rotarod (Ugo Basile, Varese, Italy) for 1 minute (min) at 2 rpm. The rotarod was then smoothly accelerated from 2 rpm to 20 rpm within 1 min. For fixed rotarod experiments, the rotarod was accelerated from 2 rpm to 10 rpm and maintained at this speed until the animal fell off. The mean latency to fall from the rotarod after 3 trials for each animal was analyzed.

### Statistical Analyses

Data are presented as means ± standard deviations (SD). Comparisons between the two groups were conducted using One-way or Two-way ANOVA with Bonferroni post-tests for time course studies or Student’s unpaired *t*-tests for end point analyses. All analyses were performed using Prism 5 (GraphPad Software, La Jolla, CA).

## Results

### MR mice on HFD have Lower Body Weight Gain Despite Increased Energy Intake

**Figure 1:**
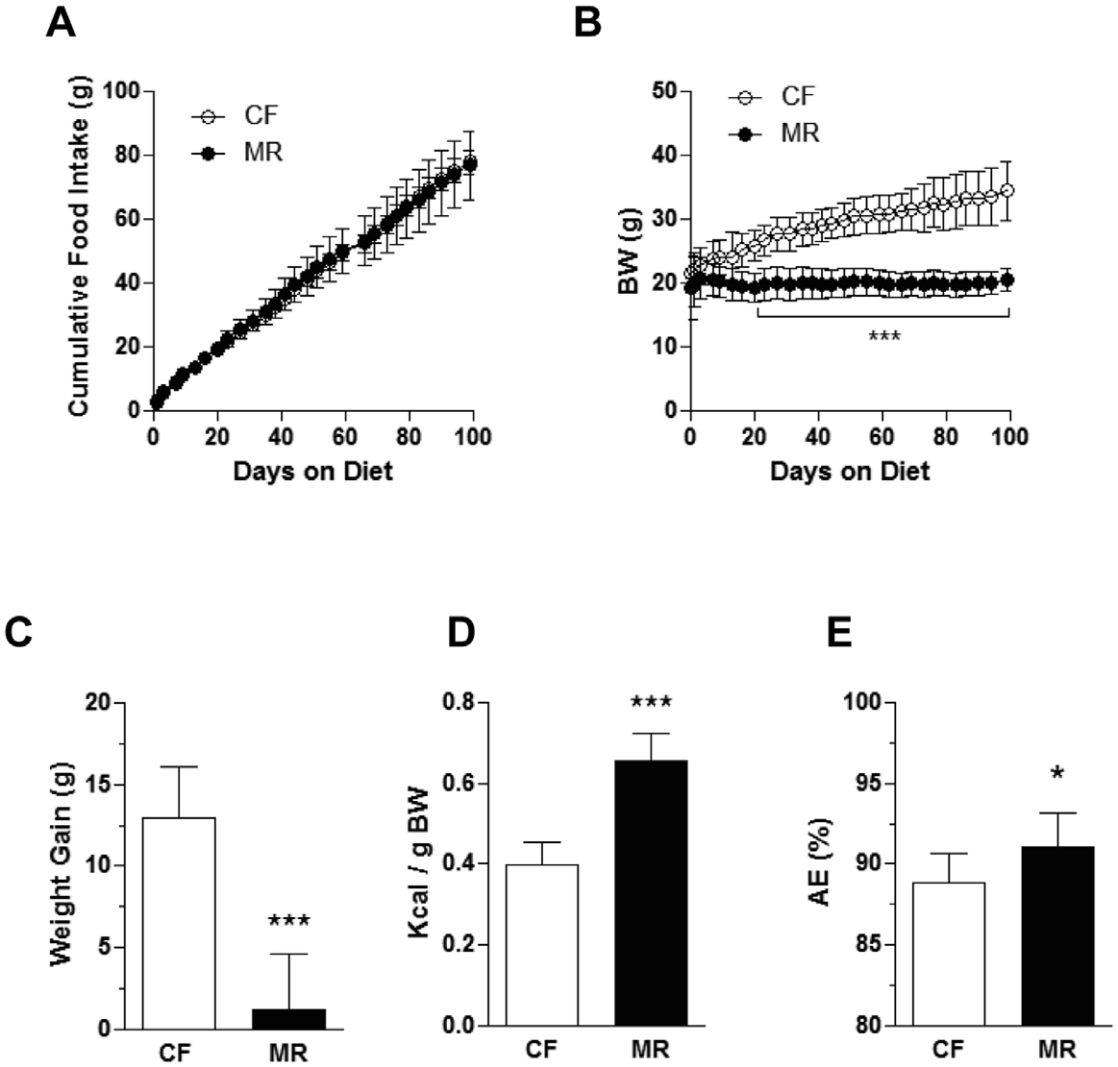
MR mice on HFD have lower body weight gain despite increased energy intake. (**A**) Cumulative food intake was measured on HFD mice twice a week for 99 days. (**B**) Body weights (BW) were measured on HFD mice twice a week for 99 days. (**C**) Body weight gain was the difference between the weights at the beginning and at the end of the study. (**D**) Energy intake was calculated based on the average daily energy (kcal) intake per gram body weight. (**E**) Absorption efficiency was estimated based on the amount of food intake and fecal output within a 24 h period as described in the Methods section. Data is presented as the mean ± SD of 8 mice per treatment group and analyzed by Two-way ANOVA followed by Bonferroni post-tests (A and B) or Student’s unpaired *t*-test (C–E). *p<0.05, ***p<0.001.

To confirm whether rodents in MR diet are obesity resistant, C57BL/6J mice were fed CF or MR diets containing high fat. This mouse strain was chosen because it is highly prone to diet-induced obesity and diabetes [3]. Cumulative food consumption was similar in both CF and MR mice on HFD throughout the study (Fig. 1A). MR mice on HFD, however, had significantly lower body weight compared to CF mice on HFD (p<0.01) 16 days after the initiation of the diet and throughout the study (day 20–day 99; p<0.001, Fig. 1B). In addition, a significant inhibition of body weight gain was observed in the MR mice on HFD (90% lower, p<0.001, Fig. 1C). When caloric intake was normalized to body weight, a 64% increase in energy intake was observed in the MR mice on HFD compared to the CF counterparts (p<0.001, Fig. 1D). In addition, a separate group of animals that was fed LFD showed similar metabolic parameters (Figs. S1A–S1D). These data are in agreement with previous reports showing the inhibition of body weight gain coupled with increased energy intake in response to MR in rats and mice [5], [6], [8], [19], [20]. Analysis of plasma lipids showed that TG levels were 27% lower in MR mice on HFD compared to the CF counterparts (p<0.05) while the TC, LDL, HDL and apo B levels were similar in both feeding groups (Table 3). Additionally, the plasma lipid levels in MR mice fed LFD were lower compared to the CF counterparts (Table S2).

**Table 3:** Blood biochemistry of HFD-fed CF and MR mice.

*Lipid Profile*	CF on HFD	MR on HFD
Triglycerides (mg/dl)	78.31±19.44	57.13±5.62*
Total Cholesterol (mg/dl)	142.51±32.49	157.29±44.47
LDL (mg/dl)	27.67±21.10	38.00±25.54
HDL (mg/dl)	102.51±18.57	110.99±23.38
Apo B (µg/ml)	29.4±4.9	31.3±5.2
*Hormone Levels*		
Adiponectin (ng/ml)	3.51±0.75	5.90±0.90***
FGF21 (pg/ml)	47.39±11.99	768.60±251.37***
IGF-1 (pg/ml)	533.75±101.82	293.24±51.03***
Leptin (pg/ml)	2140.90±966.26	100.70±28.75**

Eight weeks old C57BL/6J mice were weight-matched and fed control fed (CF) on HFD (n = 7–8) and methionine-restricted (MR) on HFD (n = 7–8) diets for 14 weeks. Data are expressed as means ± SD and compared using Student’s unpaired *t*-test.

* p<0.05,

** p<0.01,

*** p<0.001.

To examine whether the mice had complications from the absorption of the diets, food intake and fecal output was measured within a 24 h period. A significant increase in absorption efficiency was observed in MR mice compared to the CF counterparts suggesting improved absorption of the diets (Fig. 1E and Fig. S1E). Overall, these data showed that MR animals did not gain weight despite having increased energy intake and absorption efficiency.

The organ weights of MR mice on HFD were significantly lower compared to the CF mice (Table 4). Relative to total body weight (BW), perigonadal fat pads were 53% smaller (p<0.01) while hearts were 23% larger (p<0.05) in MR mice on HFD compared to CF mice on HFD. No differences in the ratios of the liver, kidney and spleen were observed between the 2 groups. Similar tissue weight differences were also observed in CF and MR mice fed LFD (Table S3).

**Table 4:** Sample weights and ratios of HFD-fed CF and MR mice.

	CF on HFD		MR on HFD	
	Weight (g)	Organ to BW Ratio (%)	Weight (g)	Organ to BW Ratio (%)
Body Weight (BW)	35.01±4.98		19.98±1.90***	
Liver	1.077±0.150	3.09±0.27	0.668±0.079***	3.36±0.41
Perigonadal Fat	1.635±0.872	4.44±1.90	0.420±0.109***	2.08±0.34**
Spleen	0.089±0.013	0.26±0.05	0.059±0.007***	0.30±0.02
Heart	0.155±0.016	0.45±0.07	0.106±0.012***	0.53±0.06*
Kidney	0.493±0.038	1.43±0.19	0.257±0.037***	1.28±0.08

Eight weeks old C57BL/6J mice were weight-matched and given control-fed (CF) on HFD (n = 8) and methionine-restricted (MR) on HFD (n = 8) diets for 14 weeks. Data are expressed as means ± SD and compared using Student’s unpaired *t*-test.

* p<0.05,

** p<0.01,

*** p<0.001.

### MR mice on HFD have Improved Glucose Homeostasis

**Figure 2:**
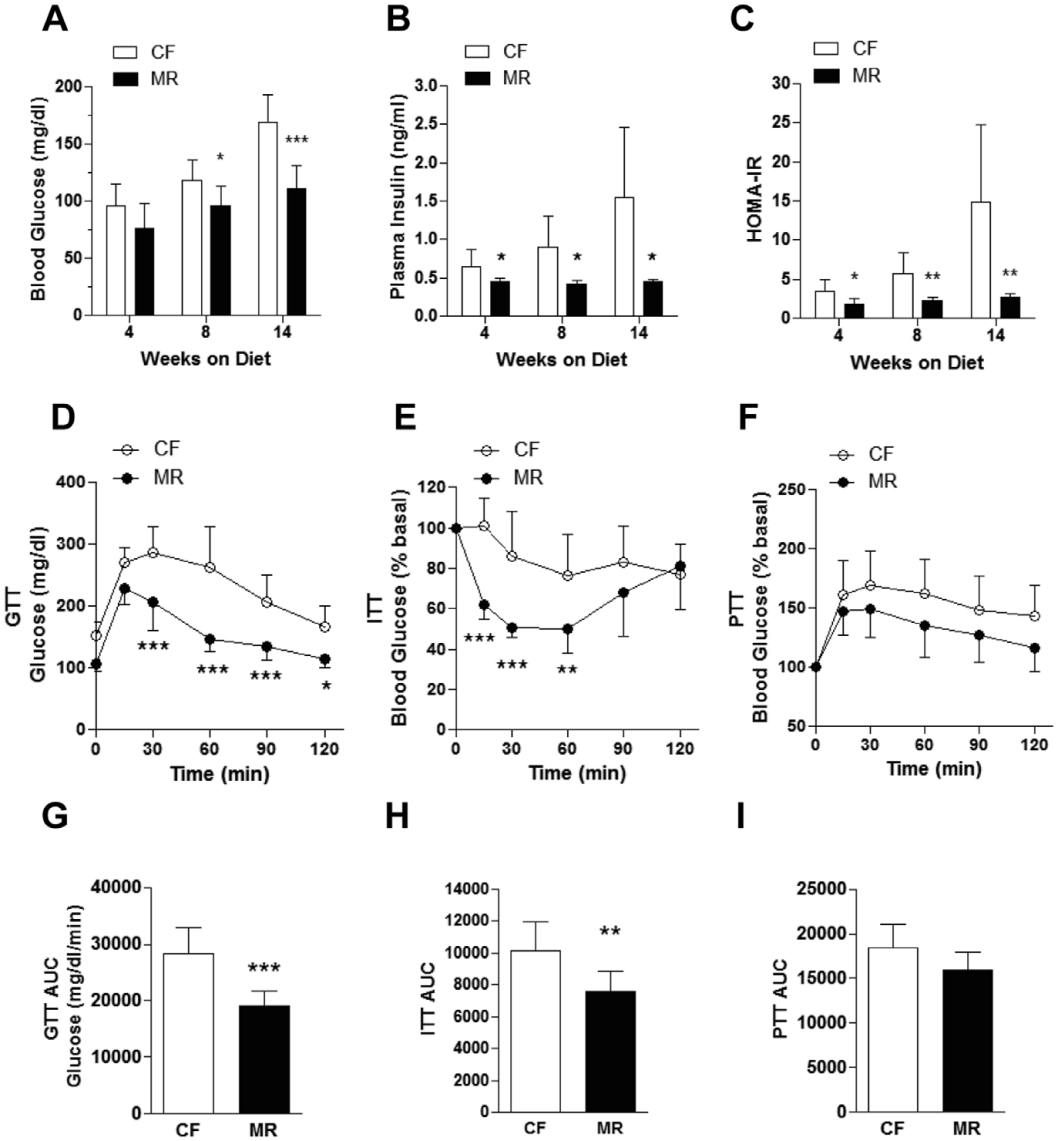
MR mice on HFD have improved glucose homeostasis. (**A**) Six hour fasting blood glucose was measured from a tail snip of each mouse using a handheld glucometer. (**B**) Fasting plasma insulin levels were measured using an ELISA kit as described in the Methods section. (**C**) Homeostasis model for insulin resistance (HOMA-IR) from HFD mice was calculated as described in the Methods section. (**D**) Intraperitoneal glucose tolerance test (GTT) was conducted on HFD mice after 8 weeks on the experimental diets. (**E**) Intraperitoneal insulin tolerance test (ITT) was conducted on HFD mice after 10 weeks on the diets. (**F**) Intraperitoneal pyruvate tolerance test (PTT) was conducted on HFD mice after 12 weeks on the diets. Areas under the curve (AUC) of GTT (**G**), ITT (**H**) and PTT (**I**). Data is presented as the mean ± SD of 8 mice per treatment group and analyzed by Two-way ANOVA followed by Bonferroni post-tests (**D–F**) or Student’s unpaired *t*-test (**A–C** and **G–I**). *p<0.05, **p<0.01, ***p<0.001.

Since the MR mice had reduced body weight gain and that HFD feeding is associated with increased risk of insulin resistance [21], the effects of MR on glucose homeostasis were examined. To assess whether HFD affected the glucose metabolism, fasting glucose levels were first determined in mice fed LFD and HFD. After 14 weeks on the diets, CF mice fed LFD had significantly lower fasting glucose levels compared to the CF mice on HFD (121.24±12.87 mg/dl vs. 169.38±24.25 mg/dl, p<0.001 by 2-way ANOVA, n = 7–8/group; Fig. 2A and Fig. S2A). While fasting plasma insulin levels in CF mice fed LFD were similar to those in CF mice on HFD (Fig. 2B and Fig. S2B), the HOMA indexes for the CF mice fed LFD were significantly lower compared to the CF mice on HFD (8.67±3.29 vs. 14.93±9.78, p<0.001 by 2-way ANOVA; Fig. 2C and Fig. S2C). These data suggest that CF mice on HFD reduced glucose tolerance and increased risk for insulin resistance than CF mice on LFD. In contrast, fasting glucose, insulin and HOMA indexes were similar in the MR mice fed LFD and the MR mice on HFD (Figs. 2A–2C and Figs. S2A–S2C). More importantly, the fasting glucose levels were significantly lower in the MR mice on HFD starting at 8 weeks on the diet until the end of the study compared to the CF counterparts (Fig. 2A). Fasting plasma insulin levels and HOMA-IR indexes were also significantly lower in the MR mice on HFD throughout the course of the study compared to the CF counterparts (Figs. 2B and 2C, respectively). GTT performed after 8 weeks on the diet showed improved glucose clearance in the MR mice on HFD compared to the CF counterparts (Figs. 2D and 2G). An ITT test at 10 weeks also showed improved insulin sensitivity in the MR animals on HFD compared to the CF counterparts (Figs. 2E and 2H). To evaluate whether hepatic glucose production was affected in both groups, a PTT was conducted at 12 weeks on the diet. MR and CF mice on HFD had similar rates of hepatic glucose production (Figs. 2F and 2I). It is important to note that the CF and MR mice on LFD were both glucose tolerant, insulin sensitive, and had similar hepatic glucose production (Figs. S2D–S2I). Overall, these results suggest that MR mice fed HFD were protected from developing insulin resistance.

### Plasma Hormone Levels in MR mice on HFD Suggest Improved Insulin Sensitivity

Since the MR mice on HFD showed improved glucose homeostasis, plasma levels of hormones associated with insulin resistance were measured. First, the CF mice on HFD presented significantly decreased adiponectin and FGF21 plasma levels compared to the CF mice on LFD (3.51±0.75 ng/ml vs. 4.58±0.41 ng/ml and 47.99±11.39 pg/ml vs. 163.96±54.77 pg/ml, p<0.01 by Student’s unpaired t-test, respectively, Table 3 and Table S2) suggesting insulin resistance due to HFD. More importantly, as shown in Table 3, adiponectin and FGF21 were significantly increased by 1.7- and 16-fold, respectively, in the MR mice on HFD compared to the CF counterparts. In contrast, IGF-1 and leptin were significantly decreased by 50% and 95%, respectively in the MR mice on HFD compared to the CF counterparts. This hormone profile suggests improved insulin sensitivity in the MR mice on HFD.

Plasma AST levels were similar in both HFD fed cohorts (CF = 73.25±10.36 IU/L vs. MR = 90.29±18.98 IU/L). Although ALT levels were significantly higher in the MR mice on HFD (CF = 24.25±3.45 IU/L vs. MR = 34.57±6.60 IU/L, p<0.01), these values are within the normal range for C57BL/6J mice on HFD according to the Mouse Phenome Database [22].

### MR mice on HFD do not Develop Hepatic Steatosis

**Figure 3:**
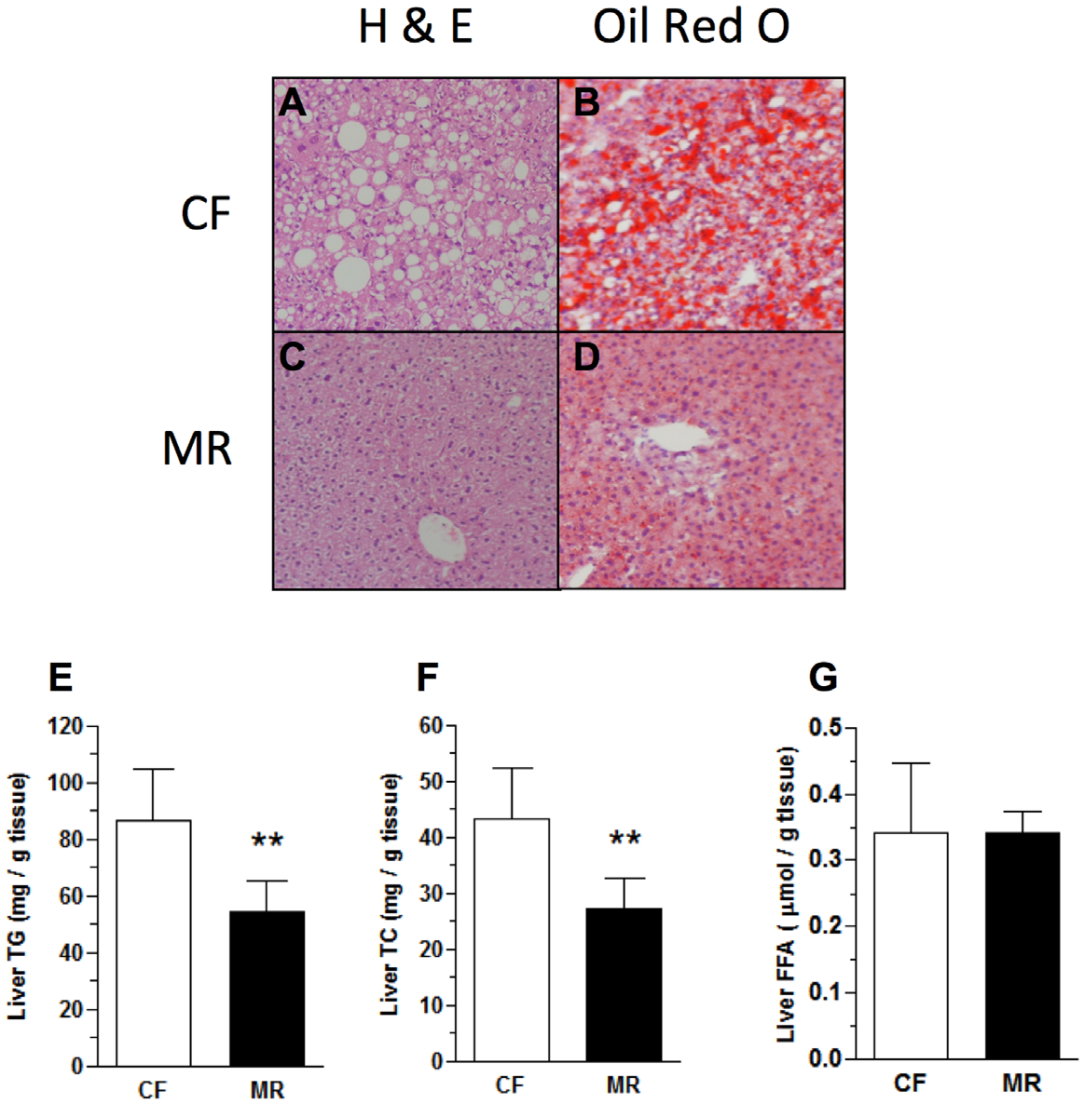
MR mice on HFD did not develop hepatic steatosis. H & E staining of liver sections from CF (**A**) and MR (**C**) mice on HFD. Oil red O staining of liver sections from CF (**B**) and MR (**D**) mice on HFD. Sections for both stains were at 5 µm and were photographed at ×100 magnification. (**E**) Liver TG levels from HFD mice. (**F**) Liver TC levels from HFD mice. (**G**) Liver FFA levels from HFD mice. All data are expressed as the mean ± SD (n = 7–8 mice per feeding group) and analyzed by Student’s unpaired *t*-test. *p<0.05, ***p<0.001.

Since HFD adversely affects hepatic lipid metabolism which could subsequently increase the risk for insulin resistance [23], the effects of MR on the accumulation of hepatic lipids were examined. Histological analyses of H & E and Oil red O staining showed increased lipid accumulation in livers from CF mice on HFD compared to MR mice on HFD (Figs. 3A–3D). The observations were supported by lipid extraction experiments which showed that MR mice on HFD had significantly lower hepatic TG (37%, p<0.01) and TC (37%, p<0.01) levels, but not FFA levels, as compared to the CF counterparts (Figs. 3E–3G). These data suggest that MR prevented the development of hepatic steatosis in mice despite feeding on a HFD.

### Hepatic Gene Expression Analyses of MR mice on HFD Correspond with Improved Insulin Sensitivity

**Figure 4:**
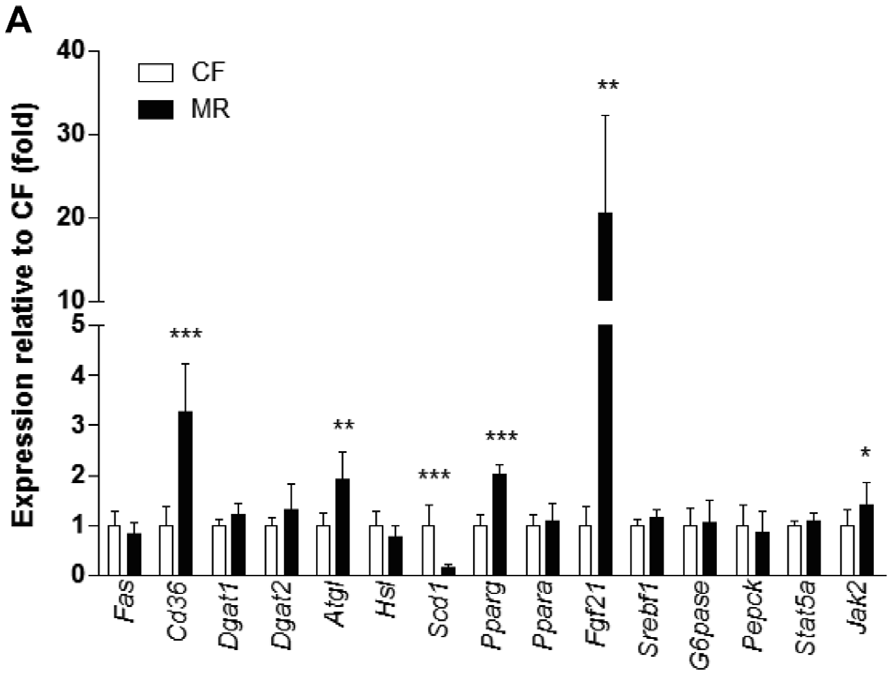
Hepatic gene expression of MR mice on HFD corresponded with improved insulin sensitivity. (**A**) Hepatic gene expression analysis by Taqman qPCR using β-actin as the housekeeping gene. All data are expressed as the mean ± SD (n = 7–8 mice per feeding group) and analyzed by Student’s unpaired *t*-test. *p<0.05, ***p<0.001.

Gene expression profiling in liver tissue by qPCR was used to gain insight about the potential molecular mechanism behind the improved glucose tolerance and hepatic lipid accumulation in MR mice on HFD (Fig. 4). Consistent with reports using MR rats, *Scd1* gene expression was downregulated (0.85-fold, p<0.001) in liver from MR mice on HFD [7]. Upregulation of fibroblast growth factor 21 (*Fgf21*, 20-fold, p<0.01), janus kinase 2 (*Jak2*, 1.4-fold, p<0.05), and *Pparγ* (1.02-fold, p<0.001) along with its target genes, adipose triglyceride lipase (*Atgl*, 0.92-fold, p<0.01) and the gene encoding fatty acid transport, cluster of differentiation 36 (*Cd36*, 2.3-fold, P<0.001) was observed in MR mice on HFD. No changes were observed for fatty acid synthase (*Fas*), diacylglyceride acyltransferases 1 and 2 (*Dgat1* and *Dgat2*), hormone sensitive lipase (*Hsl*), *Pparα*, sterol regulatory element binding protein 1 (*Srebp1*), glucose-6-phosphatase (*G6pase*), signal transducer and activator of transcription 5a (*Stat5a*) and phosphoenolpyruvate carboxykinase (*Pepck*) transcripts in both groups. These data suggest that MR mice on HFD are insulin sensitive probably due to enhanced *Fgf21* and *Pparγ* signaling activity.

### MR mice on HFD had Stunted Growth and Lower Bone Mass

**Figure 5:**
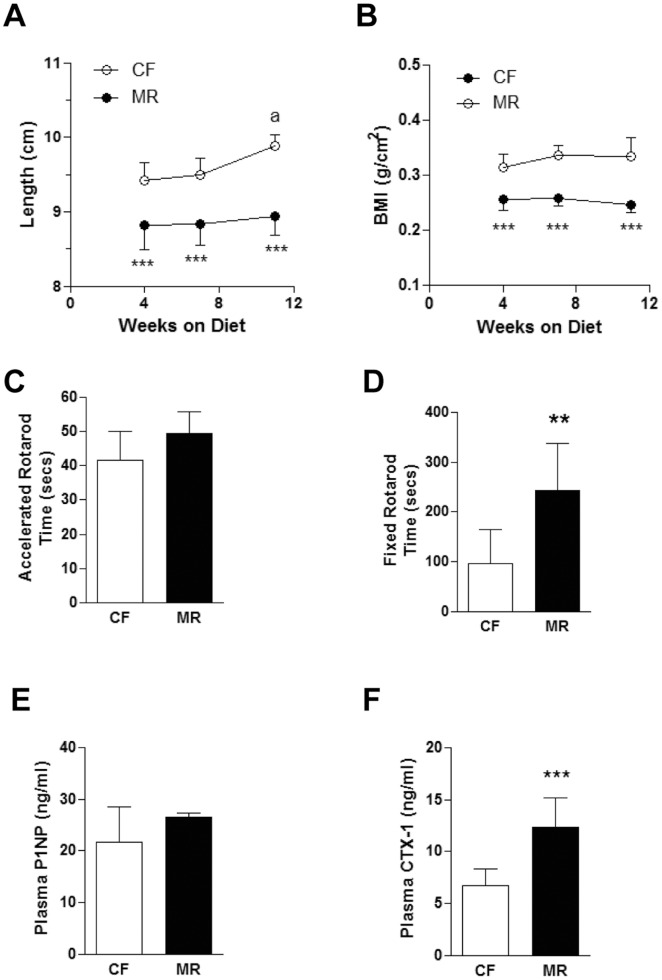
MR mice on HFD were shorter and had lower bone mass. (**A**) Mouse measurements from the tip of the nose to the base of the tail. (**B**) Mouse body mass indexes (BMI) based on the calculations mentioned in the Methods section. Statistical analysis used was One-way ANOVA followed by Bonferroni post-tests where ***p<0.001 is for CF vs MR at all time points and^ a^p<0.01 is the comparison between CF measurements at 11 and 4 weeks. (**C**) Accelerated rotarod experiments were conducted as described in the Methods section. (**D**) Fixed rotarod experiments were conducted as described in the Methods section. (**E**) Plasma N-terminal propeptide of type 1 procollagen (P1NP) as determined by ELISA. F. Plasma C-terminal telopeptide of type 1 collagen (CTX-1) as determined by ELISA. Data are expressed as the mean ± SD (n = 7–8 mice per feeding group) and Figs. C–F were analyzed by Student’s unpaired *t*-test. *p<0.05, **p<0.01, ***p<0.001.

Similar to a previous report on rats (5), MR mice on HFD not only had reduced body weight gain but were also smaller in size. In fact, MR mice on HFD were significantly shorter in length and had lower BMI compared to the CF counterparts (Fig. 5A and 5B). To determine whether motor coordination in the MR mice on HFD was affected, rotarod experiments were conducted. No difference in motor coordination was found in accelerating rotarod experiments in both dietary groups (Fig. 5C). This was supported by the fact that the MR mice on HFD weighed less and, therefore, were able to stay on a fixed rotarod for a significantly longer duration of time (CF = 97.50±66.86 seconds vs. MR = 243.57±93.68 seconds, p<0.01) than their CF counterparts (Fig. 5D). These data suggest that, despite the smaller stature of the MR mice on HFD, motor coordination is not affected.

Because MR mice on HFD were shorter in length compared to the CF counterparts, bone quality was examined. Indeed, as shown in Table 5, the left femurs from MR mice on HFD were 5% (p<0.01) shorter than those of CF counterparts. In addition, the diameters of mediolateral (8% reduced, p<0.01), anteroposterior (13% reduced, p<0.01) as well as the third trochanter shafts (14% reduced, p<0.01) were significantly smaller compared with those of the CF counterparts. Moreover, bone mineral density tests using DEXA showed that the MR mice on HFD had lower BMD and BMC (17% reduced, p<0.01 and 26% reduced, p<0.01, respectively).

**Table 5:** Bone parameters of femurs from HFD-fed CF and MR mice.

Measurements	CF on HFD	MR on HFD
Femur length (mm)	16.11±0.37	15.39±0.45**
Mediolateral shaft diameter(mm)	2.01±0.05	1.85±0.13**
Anteroposterior shaft diameter(mm)	1.31±0.10	1.14±0.07**
Third trochanter diameter (mm)	2.80±0.13	2.41±0.31**
BMD (g/cm^2^)	0.060±0.005	0.050±0.005**
BMC (g)	0.029±0.004	0.022±0.004**

Femurs collected from eight weeks old C57BL/6J mice that were weight-matched and given control-fed (CF) on HFD, (n = 8) or methionine-restricted (MR) on HFD (n = 7) diets for 14 weeks. Data are expressed as means ± SD and compared using Student’s unpaired *t*-test.

* p<0.05,

** p<0.01,

*** p<0.001.

Considering the differences in bone dimensions and density in both cohorts, possible MR effects on bone remodeling were examined. Specifically, since the bone is composed of 95% type 1 collagen [24], the presence of collagen turnover biomarkers in the plasma was examined. Levels of the collagen synthesis marker, P1NP, were similar for the 2 feeding groups while levels of the collagen degradation marker, CTX-1, were significantly higher in the MR animals on HFD by 83% (p<0.001, Figs. 5E and 5F, respectively). Overall, these data suggest that MR mice on HFD exhibited bone growth restriction in part due to increased type 1 collagen degradation.

## Discussion

The current study demonstrated for the first time that MR C57BL/6J mice on HFD are protected from developing obesity despite having increased energy intake and food absorption. These MR mice on HFD also remain glucose tolerant and insulin sensitive, effects possibly associated with increased adiponectin and hepatic FGF21 gene and protein expression. In addition, growth in MR mice on HFD was restricted without affecting motor coordination but there was a concomitant decrease in bone mass.

MR mice on HFD showed decreased body weight gain despite exhibiting hyperphagia as previously reported for rats and mice fed low fat diets [6], [7], [25]. Although hyperphagia could be attributed to the increased glutamic acid in the diets to compensate for the reduced methionine concentrations, it has been shown that serum glutamate was not increased in MR rats [7]. Moreover, the MR effects in rats could be reversed by cysteine supplementation which verified that the observed effects are specific to sulfur-containing amino acids [7].

Absorption efficiency of the diet was also improved in the MR mice on HFD compared to CF counterparts. This effect of MR on mice fed HFD could be explained by stimulation of β-adrenergic receptor causing increased energy expenditure and fat oxidation, as previously reported [26]–[28].

The effect of MR on the body composition of C57BL/6J mice corresponded with the observed increase of plasma adiponectin and the decrease of IGF-1, insulin and leptin levels [5], [19], [29]. The MR mice on HFD in this study showed increased adiponectin levels by 68% which has also been reported to potentiate insulin sensitizing effects through the activation of PPARγ signaling [30], [31]. Plasma leptin levels are associated with adipose tissue mass [32], and this study showed that the percent of perigonadal fat in MR mice on HFD was 64% smaller compared to its CF counterparts, which could explain the 95% reduction of plasma leptin levels (Table 3). Interestingly, MR mice on HFD also had a 16-fold increase in plasma levels of FGF21 compared to their CF counterparts. FGF21 is a recently discovered hormone with potent effects on glucose homeostasis [9]. Taken together, the hormone profile in MR mice on HFD is characteristic of insulin sensitive animals.

Improvement of glucose homeostasis and delayed onset of type 2 diabetes was followed by monitoring of glucose and insulin levels during the course of the study. MR mice on HFD did not present fasting hyperglycemia and hyperinsulinemia and showed an improved HOMA-IR index. This data agrees with reports on aged MR mice that showed lower glucose and insulin levels compared to aged CF mice [19], [25]. MR mice on HFD also exhibited improved glucose tolerance and peripheral insulin sensitivity without affecting hepatic glucose production. The similar expression levels of hepatic gluconeogenic genes, *G6pase* and *Pepck*, could partly explain similar hepatic glucose production in both groups. These data are also in agreement with reports on aged MR rats that had improved insulin sensitivity compared to aged CF rats [5]. It is important to mention that despite pyroglutamic acid feeding to diabetic rats and mice alleviates the symptoms of type 2 diabetes [33] and that the nitrogen balance of our diets was maintained by increasing levels of glutamic acid, the observed MR effects in this study are due to reduced levels of sulfur amino acids since these effects are reversed by cysteine supplementation of the MR diet in rats [7].

Insulin resistance is characterized by the accumulation of intrahepatic lipids due to the imbalances in the input, oxidation, synthesis and output of free-fatty acids by the hepatocytes [4], [23], [34]. Our studies showed that the MR mice on HFD had decreased accumulation of hepatic lipids as well as a hormone profile that promotes insulin sensitivity. In addition, the hepatic gene expression data agreed with reports showing the upregulation of *Pparγ* in MR rats [7] and its target genes: *Cd36* and *Atgl*. In fact, rosiglitazone, a *Pparγ* agonist, was reported to ameliorate hepatic steatosis and improve insulin sensitivity [16], [35]–[38]. However, this is in contrast with studies showing hepatic overexpression of *Pparγ* in mouse models lead to adipogenic lipogenesis [39]–[41] and that *Pparγ* and *Cd36* are upregulated during hepatic steatosis [42]–[44]. Nevertheless, decreased in hepatic *Scd1* gene expression in this model coincided with observations in MR rats [7] suggesting increased β-oxidation [45]. These data, therefore, suggest that MR mice on HFD are protected from developing hepatic steatosis which could attenuate type 2 diabetes.

Another plausible explanation for the protection against insulin resistance by MR on mice fed HFD could be on the effects of FGF21. MR mice on HFD showed a 20-fold upregulation of hepatic *Fgf21* gene expression which corresponded with a 16-fold increase of plasma FGF21. Therapeutic administration of FGF21 in diabetic rodent models and overexpression of FGF21 in mice were previously shown to improve glucose homeostasis [9], [46]. In contrast, Fisher *et al.* reported that obese mice have increased hepatic mRNA and circulating plasma FGF21 levels compared to lean mice, but this was attributed to FGF21 resistance and reduced glucose clearance rate [47]. Xu *et al.* however showed that injecting recombinant FGF21 into obese mice reversed hepatic steatosis, increased energy expenditure and improved insulin sensitivity [48]. These observations were partly explained by Dutchak *et al.* who reported that white adipose tissue of FGF21-deficient mice had a decrease in PPARγ activity [10]. In the latter studies, it was proposed that FGF21 enhanced PPARγ activity in white adipose tissue by suppressing PPARγ sumoylation, which leads to improved insulin sensitivity [10]. It is also likely that, in mice fed HFD, FGF21 could be exerting an important role in the liver where MR led to the transcriptional upregulation of *Pparγ* gene expression which correlated with improved glucose homeostasis and decreased hepatic steatosis. Thus, the robust effects of MR on FGF21 could possibly explain, at least in part, the protection against type 2 diabetes in HFD mice.

MR mice on HFD presented stunted growth and reduced bone density. Although we did not observe any adverse effect on the motor function in the MR mice on HFD, the plasma biomarker for type 1 collagen degradation, CTX-1, was increased compared to the CF mice on HFD. Serum CTX-1 has been reported to be a specific marker for bone resorption [49]. The growth restriction observed in the MR mice could be further explained by the effect of MR on FGF21 [50] and its subsequent effects on the growth hormone [51]. De Sousa-Coelho *et al.* reported that amino acid deprivation in mouse liver and HepG2 cells induced FGF21, a target of activating transcription factor 4 (ATF4) [50]. In addition, Inagaki *et al*. showed that FGF21 transgenic animals had decreased levels of plasma IGF-1 and were smaller than the wild-type counterparts [51]. These investigators also showed that the effects of FGF21 were mediated by a decrease in phosphorylated Stat5 and an increase in Jak2 phosphorylation [51]. Although hepatic *Stat5a* gene expression was not affected in our studies, *Jak2* gene expression was increased in MR mice on HFD. Therefore, our data could also suggest that FGF21 interrupts growth hormone signaling downstream of Jak2. Furthermore, Wei *et al.* showed that, compared to their wild-type littermates, transgenic mice overexpressing FGF21 had lower BMD, which was associated with the upregulation of *Pparγ2* in osteoblasts and increased levels of urinary and serum CTX-1 [11]. Rosiglitazone, a PPARγ agonist, increased bone loss in the wild-type mice but had no additive effect on FGF21 knockout mice suggesting that FGF21 could be causing decreased bone mass via PPARγ signaling [11]. Taken together, decreased bone mass in MR mice on HFD could be explained, at least in part, by enhanced FGF21 activity. It is, however, important to mention that the mice used in our experiments were 8 weeks old at the beginning of the studies and had not reached their peak growth. Therefore, studies using aged mice that have reached maximum growth as well as young mice with fast growth rates are necessary to elucidate the effects of MR on the bone remodeling.

In conclusion, studies using MR mice on HFD confirmed that MR protects rodents from developing obesity, insulin resistance and type 2 diabetes, conditions that are observed during aging. The MR effects may be associated with the reduction of hepatic lipid accumulation and favorable hormonal changes associated with insulin sensitivity. MR significantly upregulated the expression of FGF21 at the gene and protein levels and its enhanced activity is proposed to be involved in the bone remodeling changes observed in MR mice on HFD. Overall, these results reveal beneficial and unfavorable effects of MR.

## Supporting Information

Figure S1 **MR mice on LFD have lower body weight gain despite increased energy intake.** (**A**) Cumulative food intake was measured on LFD mice twice a week for 99 days. (**B**) Body weights (BW) were measured on HFD mice twice a week for 99 days. (**C**) Body weight gain was the difference between the weights at the beginning and at the end of the study. (**D**) Energy intake was calculated based on the average daily energy (kcal) intake per gram body weight. (**E**) Absorption efficiency was estimated based on the amount of food intake and fecal output within a 24 h period as described in the Methods section. Data is presented as the mean ± SD of 8 mice per treatment group and analyzed by Two-way ANOVA followed by Bonferroni post-tests (A and B) or Student’s unpaired *t*-test (C–E). *p<0.05, ***p<0.001.
(TIF)

Figure S2 **Glucose homeostasis for the CF and MR mice on LFD were similar.** (**A**) Six hour fasting blood glucose was measured from a tail snip of each mouse using a handheld glucometer. (**B**) Fasting plasma insulin levels were measured using an ELISA kit as described in the Methods section. (**C**) Homeostasis model for insulin resistance (HOMA-IR) from LFD mice was calculated as described in the Methods section. (**D**) Intraperitoneal glucose tolerance test (GTT) was conducted on LFD mice after 8 weeks on the experimental diets. (**E**) Intraperitoneal insulin tolerance test (ITT) was conducted on LFD mice after 10 weeks on the diets. (**F**) Intraperitoneal pyruvate tolerance test (PTT) was conducted on LFD mice after 12 weeks on the diets. Areas under the curve (AUC) of GTT (**G**), ITT (**H**) and PTT (**I**). Data is presented as the mean ± SD of 8 mice per treatment group and analyzed by Two-way ANOVA followed by Bonferroni post-tests (**D–F**) or Student’s unpaired *t*-test (**A–C** and **G–I**). **p<0.01, ***p<0.001.
(TIF)

Table S1 **Diet Composition of LFD-fed mice.** Low fat diets were purchased from Research Diets, Inc., New Brunswick, NJ. Control-fed (CF) on LFD catalog number: A11051302 and methionine-restricted (MR) on LFD catalog number: A11051301. Numbers in parenthesis are levels of DL-methionine and L-glutamic acid in the CF diet.
(DOCX)

Table S2 **Blood Biochemistry of CF and MR mice on LFD.** Eight weeks old C57BL/6J mice were weight-matched and fed control fed (CF) on LFD (n = 7–8) and methionine-restricted (MR) on LFD (n = 7–8) diets for 14 weeks. Data are expressed as means ± SD and compared using Student’s unpaired *t*-test. *p<0.05, **p<0.01, ***p<0.001.
(DOCX)

Table S3 **Sample weights and ratios of organs from CF and MR mice on LFD.** Eight weeks old C57BL/6J mice were weight-matched and given control-fed (CF) on LFD (n = 8) and methionine-restricted (MR) on LFD (n = 8) diets for 14 weeks. Data are expressed as means ± SD and compared using Student’s unpaired *t*-test. *p<0.05, **p<0.01, ***p<0.001.
(DOCX)
