# Correction: Methionine-Restricted C57BL/6J Mice Are Resistant to Diet-Induced Obesity and Insulin Resistance but Have Low Bone Density

**DOI:** 10.1371/journal.pone.0104050

**Published:** 2014-07-25

**Authors:** The PLOS ONE Staff

In the Abstract and in the Materials and Methods, the article states that control and MR groups were fed with 0.86% methionine or 0.12% methionine. These values are correct, but they represent KCal percentages.

The percentage values represented in Table 1 and Table S1 are gram percentages. The authors have provided corrected versions of Table 1 and Table S1 below.

**Table 1:** Diet composition of HFD-fed CF and MR mice.

Ingredients	% gm / % Kcal
L-Arginine	1.48/1.13
L-Histidine-HCl-H2O	0.44/0.33
L-Isoleucine	1.09/0.83
L-Leucine	1.47/1.1
L-Lysine	1.91/1.45
DL-Methionine	0.16/0.12 (1.14/0.86)
L-Phenylalanine	1.53/1.15
L-Threonine	1.09/0.83
L-Tryptophan	0.24/0.18
L-Valine	1.09/0.83
L-Glutamic Acid	4.55/3.45 (3.57/2.71)
Glycine	3.08/2.33
Corn Starch	0/0
Maltodextrin	7.52/5.68
Dextrose	6.62/5
Sucrose	19.85/15
Cellulose	6.62/0
Lard	28.98/49.28
Corn Oil	6.09/10.35
Minerals	4.63/0
Vitamins	1.32/1
Choline Bitartrate	0.26/0
Dye	0.01/0

High fat diets were purchased from Research Diets, Inc., New Brunswick, NJ. Control-fed (CF) on HFD catalog number: A11051306 and methionine-restricted (MR) on HFD catalog number: A11051305. Numbers in parenthesis are levels of DL-methionine and L-glutamic acid in the CF diet.

**Table S1:** Diet Composition of LFD-fed mice.

Ingredients	% gm / % Kcal	
L-Arginine	1.09/1.13	
L-Histidine-HCl-H2O	0.32/0.33	
L-Isoleucine	0.8/0.83	
L-Leucine	1.08/1.1	
L-Lysine	1.4/1.45	
DL-Methionine	0.12/0.12 (0.84/0/86)	
L-Phenylalanine	1.13/1.15	
L-Threonine	0.8/0.83	
L-Tryptophan	0.17/0.18	
L-Valine	0.8/0.83	
L-Glutamic Acid	3.34/3.45 (2.62/2.71)	
Glycine	2.26/2.33	
Corn Starch	53.38/54.95	
Maltodextrin	0/0	
Dextrose	4.86/5	
Sucrose	14.57/15	
Cellulose	4.86/0	
Lard	0/0	
Corn Oil	4.47/10.35	
Minerals	3.4/0	
Vitamins	0.97/1	
Choline Bitartrate	0.19/0	
Dye	0/0	

Low fat diets were purchased from Research Diets, Inc., New Brunswick, NJ. Control-fed (CF) on LFD catalog number: A11051302 and methionine-restricted (MR) on LFD catalog number: A11051301. Numbers in parenthesis are levels of DL-methionine and L-glutamic acid in the CF diet.

## References

[1] Ables GP, Perrone CE, Orentreich D, Orentreich N (2012) Methionine-Restricted C57BL/6J Mice Are Resistant to Diet-Induced Obesity and Insulin Resistance but Have Low Bone Density. PLoS ONE 7(12): e51357 doi:10.1371/journal.pone.0051357 23236485 10.1371/journal.pone.0051357 PMC3518083

