# Supplementary figures and images for: Methionine-Restricted C57BL/6J Mice Are Resistant to Diet-Induced Obesity and Insulin Resistance but Have Low Bone Density
Source: PLoS One. 2012 Dec 7;7(12):e51357. doi: 10.1371/journal.pone.0051357 (PMC3518083; doi:10.1371/journal.pone.0051357)

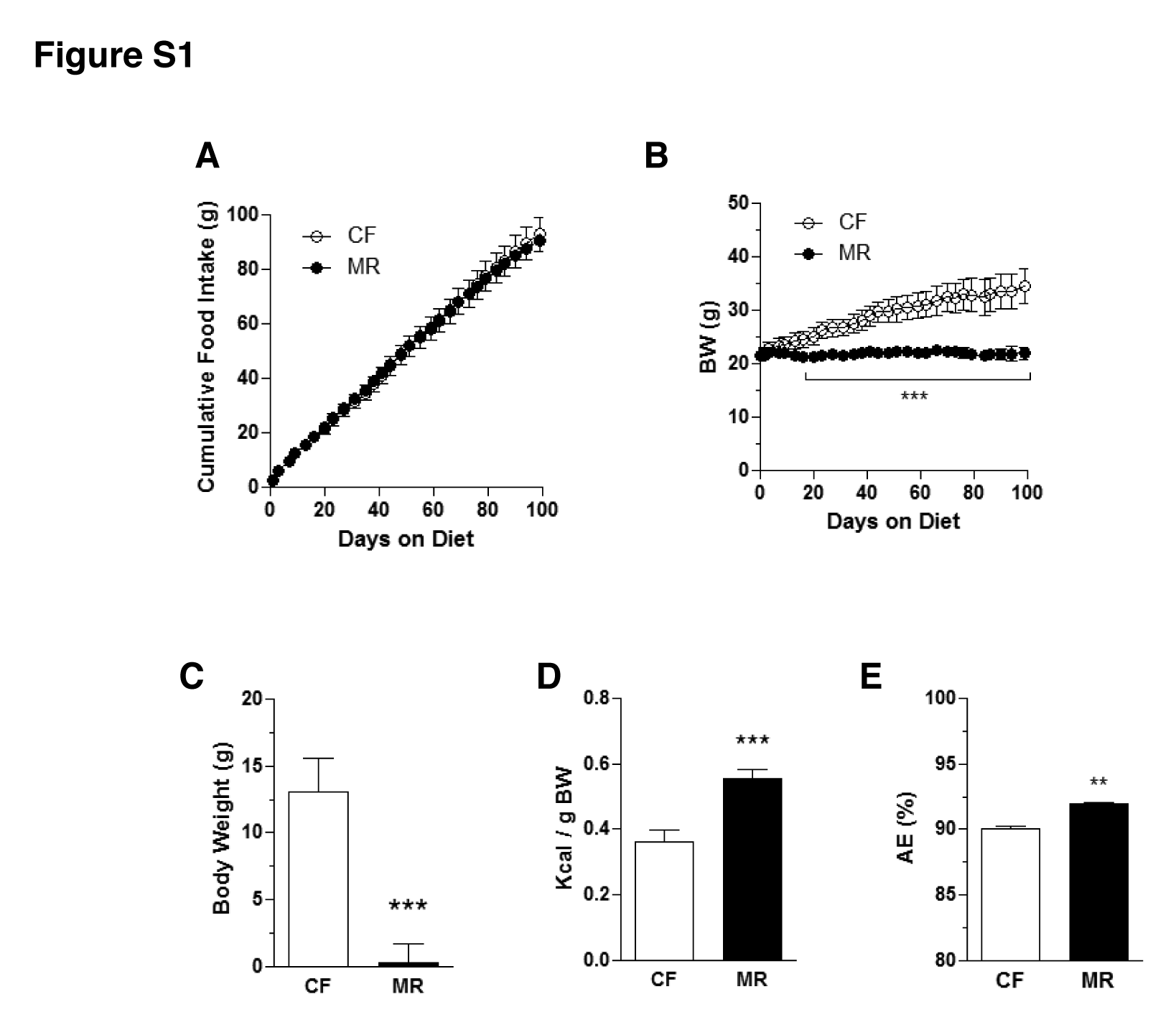

Supplement: Figure S1 — MR mice on LFD have lower body weight gain despite increased energy intake. (A) Cumulative food intake was measured on LFD mice twice a week for 99 days. (B) Body weights (BW) were measured on HFD mice twice a week for 99 days. (C) Body weight gain was the difference between the weights at the beginning and at the end of the study. (D) Energy intake was calculated based on the average daily energy (kcal) intake per gram body weight. (E) Absorption efficiency was estimated based on the amount of food intake and fecal output within a 24 h period as described in the Methods section. Data is presented as the mean ± SD of 8 mice per treatment group and analyzed by Two-way ANOVA followed by Bonferroni post-tests (A and B) or Student’s unpaired t-test (C–E). *p<0.05, ***p<0.001. (TIF) [file pone.0051357.s001.tif]

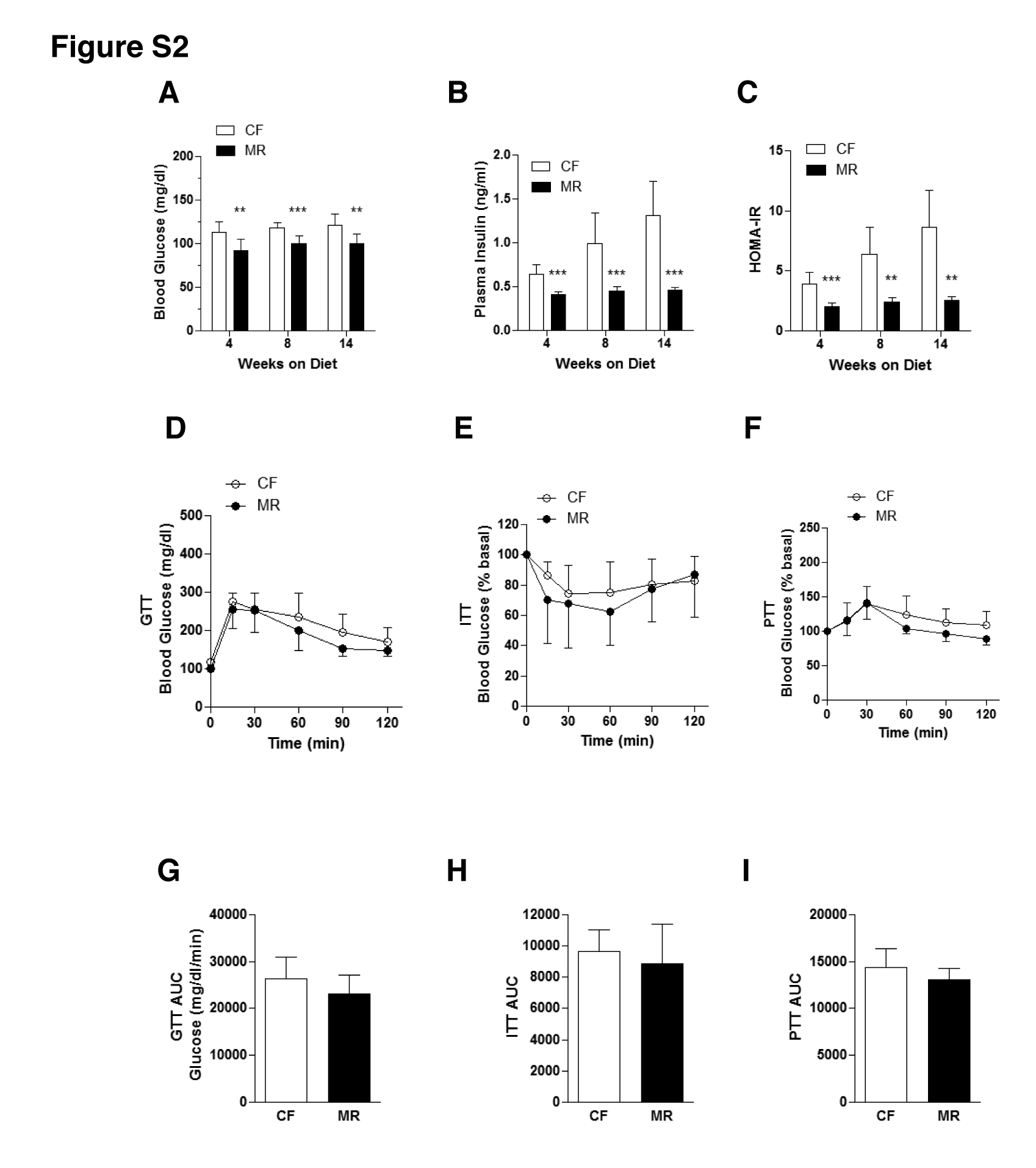

Supplement: Figure S2 — Glucose homeostasis for the CF and MR mice on LFD were similar. (A) Six hour fasting blood glucose was measured from a tail snip of each mouse using a handheld glucometer. (B) Fasting plasma insulin levels were measured using an ELISA kit as described in the Methods section. (C) Homeostasis model for insulin resistance (HOMA-IR) from LFD mice was calculated as described in the Methods section. (D) Intraperitoneal glucose tolerance test (GTT) was conducted on LFD mice after 8 weeks on the experimental diets. (E) Intraperitoneal insulin tolerance test (ITT) was conducted on LFD mice after 10 weeks on the diets. (F) Intraperitoneal pyruvate tolerance test (PTT) was conducted on LFD mice after 12 weeks on the diets. Areas under the curve (AUC) of GTT (G), ITT (H) and PTT (I). Data is presented as the mean ± SD of 8 mice per treatment group and analyzed by Two-way ANOVA followed by Bonferroni post-tests (D–F) or Student’s unpaired t-test (A–C and G–I). **p<0.01, ***p<0.001. (TIF) [file pone.0051357.s002.tif]
