# Supplementary material for: Methionine-Restricted C57BL/6J Mice Are Resistant to Diet-Induced Obesity and Insulin Resistance but Have Low Bone Density
Source: PLoS One. 2012 Dec 7;7(12):e51357. doi: 10.1371/journal.pone.0051357 (PMC3518083; doi:10.1371/journal.pone.0051357)
Supplement: Table S1 — Diet Composition of LFD-fed mice. Low fat diets were purchased from Research Diets, Inc., New Brunswick, NJ. Control-fed (CF) on LFD catalog number: A11051302 and methionine-restricted (MR) on LFD catalog number: A11051301. Numbers in parenthesis are levels of DL-methionine and L-glutamic acid in the CF diet. (DOCX) [file pone.0051357.s003.docx]

**Table S1. Diet composition of LFD-fed mice.**

|  |  |
| --- | --- |
| Ingredients (gm) | 10% Fat |
| L-Arginine | 11.2 |
| L-Histidine-HCl-H2O | 3.3 |
| L-Isoleucine | 8.2 |
| L-Leucine | 11.1 |
| L-Lysine | 14.4 |
| DL-Methionine | 1.2 (8.6) |
| L-Phenylalanine | 11.6 |
| L-Threonine | 8.2 |
| L-Tryptophan | 1.8 |
| L-Valine | 8.2 |
| L-Glutamic Acid | 34.4 (27) |
| Glycine | 23.3 |
| Corn Starch | 549.5 |
| Maltodextrin | 0.0 |
| Dextrose | 50.0 |
| Sucrose | 150.0 |
| Lard | 0.0 |
| Corn Oil | 46.0 |
| Minerals | 35.0 |
| Vitamins | 10.0 |
| Choline Bitartrate | 2.0 |

Low fat diets were purchased from Research Diets, Inc., New Brunswick, NJ. Control-fed (CF) on LFD catalog number: A11051302 and methionine-restricted (MR) on LFD catalog number: A11051301. Numbers in parenthesis are levels of DL-methionine and L-glutamic acid in the CF diet.
