# Supplementary material for: Methionine-Restricted C57BL/6J Mice Are Resistant to Diet-Induced Obesity and Insulin Resistance but Have Low Bone Density
Source: PLoS One. 2012 Dec 7;7(12):e51357. doi: 10.1371/journal.pone.0051357 (PMC3518083; doi:10.1371/journal.pone.0051357)
Supplement: Table S2 — Blood Biochemistry of CF and MR mice on LFD. Eight weeks old C57BL/6J mice were weight-matched and fed control fed (CF) on LFD (n = 7–8) and methionine-restricted (MR) on LFD (n = 7–8) diets for 14 weeks. Data are expressed as means ± SD and compared using Student’s unpaired t-test. *p<0.05, **p<0.01, ***p<0.001. (DOCX) [file pone.0051357.s004.docx]

**Table S2. Blood Biochemistry of CF and MR mice on LFD.**

|  | |  |
| --- | --- | --- |
| *Lipid Profile* | CF on LFD | MR on LFD |
| Triglycerides (mg/dl) | 72.4 ± 3.77 | 64.25 ± 9.88 |
| Total Cholesterol (mg/dl) | 186.03 ± 13.64 | 121.67 ± 16.55^***^ |
| LDL (mg/dl) | 45.4 ± 7.74 | 24.82 ± 7.09^***^ |
| HDL (mg/dl) | 126.18 ± 10.83 | 84 ± 11.56^***^ |
| Apo B (µg/ml) | 33.15 ± 4.11 | 37 ± 6.14 |
| *Hormone Levels* |  |  |
| Adiponectin (ng/ml) | 4.58 ± 0.41 | 6.35 ± 0.61^***^ |
| FGF21 (pg/ml) | 163.96 ± 54.77 | 680.93 ± 241.76^***^ |
| IGF-1 (pg/ml) | 568.24 ± 102.37 | 287.20 ± 42.86^***^ |
| Leptin (pg/ml) | 1779.52 ± 459.64 | 153.86 ± 46.56^**^ |

Eight weeks old C57BL/6J mice were weight-matched and fed control fed (CF) on LFD (n = 7-8) and methionine-restricted (MR) on LFD (n = 7-8) diets for 14 weeks. Data are expressed as means ± SD and compared using Student’s unpaired *t*-test. ^*^p < 0.05, ^**^p < 0.01, ^***^p < 0.001.
