# Supplementary material for: Methionine-Restricted C57BL/6J Mice Are Resistant to Diet-Induced Obesity and Insulin Resistance but Have Low Bone Density
Source: PLoS One. 2012 Dec 7;7(12):e51357. doi: 10.1371/journal.pone.0051357 (PMC3518083; doi:10.1371/journal.pone.0051357)
Supplement: Table S3 — Sample weights and ratios of organs from CF and MR mice on LFD. Eight weeks old C57BL/6J mice were weight-matched and given control-fed (CF) on LFD (n = 8) and methionine-restricted (MR) on LFD (n = 8) diets for 14 weeks. Data are expressed as means ± SD and compared using Student’s unpaired t-test. *p<0.05, **p<0.01, ***p<0.001. (DOCX) [file pone.0051357.s005.docx]

**Table S3. Sample weights and ratios of organs from CF and MR mice on LFD.**

|  | | |  |  |  |
| --- | --- | --- | --- | --- | --- |
|  | CF on LFD | |  | MR on LFD | |
|  | Weight (g) | Organ to BW Ratio (%) |  | Weight (g) | Organ to BW Ratio (%) |
| Body Weight (BW) | 35.44 ± 2.33 |  |  | 21.33 ± 2.04^***^ |  |
| Liver | 1.203 ± 0.096 | 3.46 ± 0.22 |  | 0.748 ± 0.031^***^ | 3.48 ± 0.19 |
| Perigonadal Fat | 1.741 ± 0.215 | 4.90 ± 0.37 |  | 0.558 ± 0.055^***^ | 2.59 ± 0.18^**^ |
| Spleen | 0.079 ± 0.005 | 0.22 ± 0.01 |  | 0.060 ± 0.011^***^ | 0.28 ± 0.05^**^ |
| Heart | 0.142 ± 0.012 | 0.40 ± 0.03 |  | 0.117 ± 0.013^***^ | 0.54 ± 0.04^**^ |
| Kidney | 0.395 ± 0.040 | 1.12 ± 0.09 |  | 0.395 ± 0.040^***^ | 1.17 ± 0.03 |

Eight weeks old C57BL/6J mice were weight-matched and given control-fed (CF) on LFD (n = 8) and methionine-restricted (MR) on LFD (n = 8) diets for 14 weeks. Data are expressed as means ± SD and compared using Student’s unpaired *t*-test. ^*^p < 0.05, ^**^p < 0.01, ^***^p < 0.001.
