# Supplementary figures and images for: Correction: Methionine-Restricted C57BL/6J Mice Are Resistant to Diet-Induced Obesity and Insulin Resistance but Have Low Bone Density
Source: PLoS One. 2026 Mar 9;21(3):e0344462. doi: 10.1371/journal.pone.0344462 (PMC12970924; doi:10.1371/journal.pone.0344462)

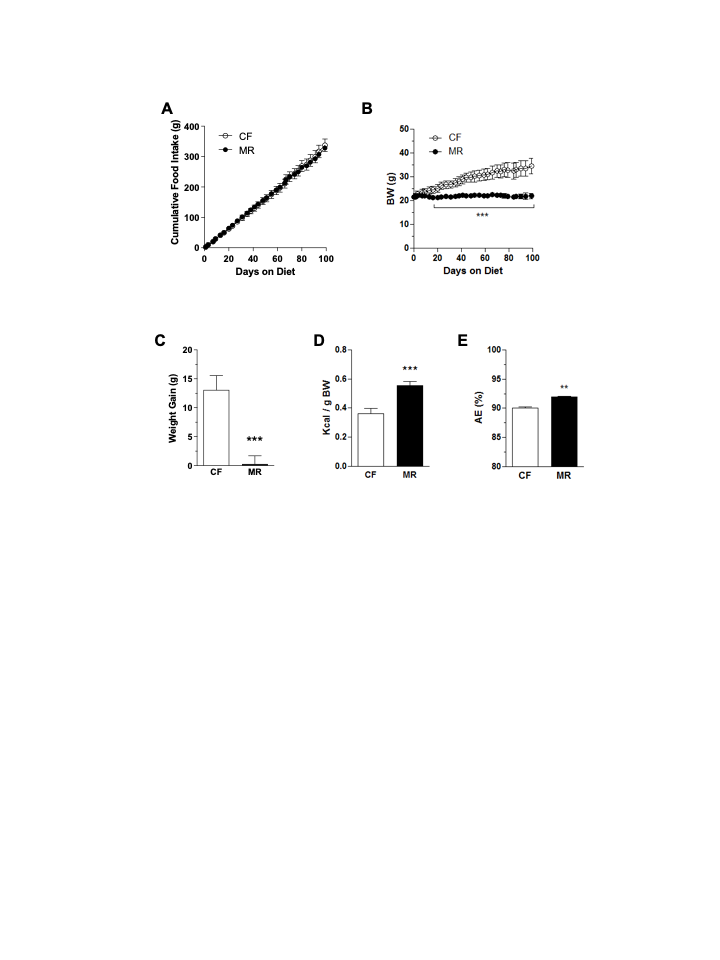

Supplement: S1 Fig — (A) Cumulative food intake was measured on LFD mice twice a week for 99 days. (B) Body weights (BW) were measured on HFD mice twice a week for 99 days. (C) Body weight gain was the difference between the weights at the beginning and at the end of the study. (D) Energy intake was calculated based on the average daily energy (kcal) intake per gram body weight. (E) Absorption efficiency was estimated based on the amount of food intake and fecal output within a 24 h period as described in the Methods section. Data is presented as the mean ± SD of 8 mice per treatment group and analyzed by Two-way ANOVA followed by Bonferroni post-tests (A and B) or Student’s unpaired t-test (C–E). *p < 0.05, ***p < 0.001. (TIFF) [file pone.0344462.s001.tiff]
